# Nightmares and childhood trauma in depression and insomnia among adolescents: A pilot study

**DOI:** 10.1192/j.eurpsy.2025.509

**Published:** 2025-08-26

**Authors:** Y. Li, H. F. Sit, Y. L. Wong, S. X. Li

**Affiliations:** 1 Department of Psychology; 2 The State Key Laboratory of Brain and Cognitive Sciences, The University of Hong Kong, Hong Kong, Hong Kong

## Abstract

**Introduction:**

Nightmares have been linked to childhood trauma and an increased risk for mental health problems, such as depression. Meanwhile, there is a high comrobdity of nightmares and insomnia. Yet relatively few studies have compared the clinical presentations of nightmares in different clinical groups. Additionally, considering the close association between childhood trauma, insomnia, and depression, there might exist potential unique interactions between childhood trauma and clinical diagnoses on nightmare experience.

**Objectives:**

This case-control study aimed to compare nightmare-related parameters (i.e., frequency, distress, severity, and impairment), and childhood trauma among adolescents with depression only (DG), insomnia only (IG), and healthy control (HG) groups. We explored the interaction between diagnosis and childhood trauma on nightmare parameters.

**Methods:**

Participants completed a clinical interview to ascertain their eligibility. Data on demographic and clinical information, childhood trauma as assessed by the childhood trauma Questionnaire (CTQ) , and nightmare-related parameters, including nightmare frequency, nightmare distress, nightmare severity, and nightmare impairment, were analysed in the current study. Analysis of variance (ANOVA) and multivariate analysis of variance (MANOVA) were used to examine the group differences, and regression analysis was used to examine the interaction effect on study variables.

**Results:**

Adolescents with insomnia (*N* = 31; age 16.84 ± 1.88 years; female: 54.8%), female: 55.2%), depression (*N* = 22; age 17.50 ± 2.18 years; female: 54.5%) and healthy controls (*N* = 31; age 16.84 ± 1.88 years; female: 54.8%) were recruited. Compared to the HG, the IG and DG had greater nightmare distress (IG: *p* = .024; DG: *p* =.005) and nightmare impairment (IG: *p* = .007; DG: *p* = .031), but not nightmare frequency. However, only DG showed significantly higher nightmare severity (*p* = .038). No other significant differences were found in nightmare parameters between IG and DG (all *p* > .05). For childhood trauma, only DG showed significantly higher scores in emotional abuse (*p* =.013), emotional neglect (*p* = .021), and physical neglect (*p* = .012). No interaction effect of childhood trauma and clinical diagnosis was found on nightmare-related parameters (all *p* > .05).

**Conclusions:**

This study showed that adolescents with insomnia or depression exhibited greater nightmare-related distress and impairment. Higher nightmare severity may be a unique characteristic in adolescents with depression but not for insomnia. Despite the depression group reporting significantly more childhood traumatic experiences, the potential interaction effect between diagnosis and childhood trauma was not observed on nightmare-related parameters. Future research may examine the relationship between the relevant variables in a larger sample size using a longitudinal design.

**Disclosure of Interest:**

Y. Li: None Declared, H. F. Sit: None Declared, Y. L. Wong: None Declared, S. X. Li Grant / Research support from: This work was funded by Seed Fund for Basic Research, The University of Hong Kong and General Research Fund (Ref. 17613820), Research Grants Council, University Grants Committee, Hong Kong SAR, China.

